# Supplementary material for: Drug repurposing for aging research using model organisms
Source: Aging Cell. 2017 Jun 16;16(5):1006–15. doi: 10.1111/acel.12626 (PMC5595691; doi:10.1111/acel.12626)
Supplement: Supplementary file 7 — Data S1 Zip‐Archive of all report cards. [file ACEL-16-1006-s007.zip › RC_CIT.pdf]

CIT

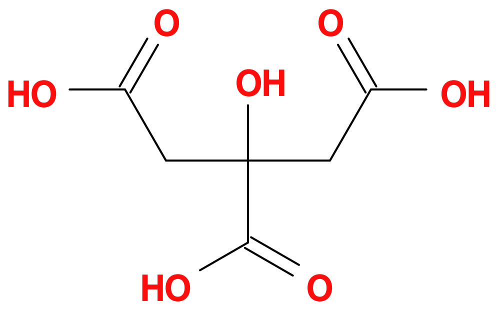

Database identifiers

ChEMBLCompound	CHEMBL1261
DrugBank	DB04272
CHEBI	30769
eMolecules	475729

Ranking

	Rank	Score
Drosophila	349/697	0.431
C. elegans	217/591	0.237

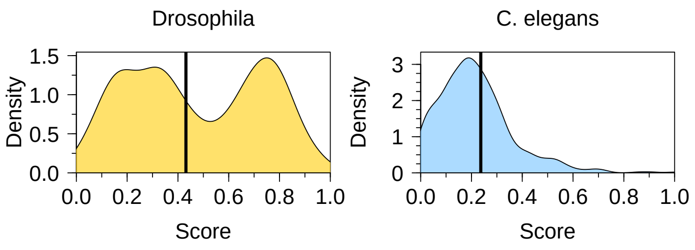

	Ageing implication	Domain conservation	Binding site conservation	Binding affinity	Bioavailability	Lipinski	Promiscuity	Purchasability	Drug approval	**Total**
Drosophila	1.0	0.947	1.0	0.299	(0.9)	0.0	-0.024	0.1	0.1	0.431
C. elegans	1.0	0.893	1.0	0.299	0.226	0.0	-0.024	0.1	0.1	0.237

Names

- **citric acid**
- 2-Hydroxy-1,2,3-propanetricarboxylic acid
- 2-Hydroxytricarballylic acid
- 3-Carboxy-3-hydroxypentane-1,5-dioic acid
- Citronensaeure
- E330
- H3cit
- Urologic G

Roles

food acidity regulator, chelator, antimicrobial agent, fundamental metabolite

Status

Approved drug (according to ChEMBL)	Yes
Number of Rule of 5 violations	0
Binding affinity to original target in log units (RF-Score prediction)	4.15
Burns *C. elegans* bioavailability prediction	-3.63

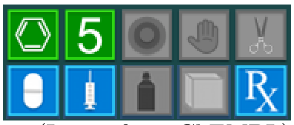

(Image from ChEMBL)

Medical Information

Toxicity: ORAL (LD50): Acute: 5040 mg/kg [Mouse]. 3000 mg/kg [Rat].

(Information from DrugBank)

Compound Target Characteristics

Macrophage migration inhibitory factor

Best gene implication in ageing for this target family came from gene P34884 annotated in UniProt release 2014_02. Annotation GO subterm of 7568 (aging) was Inferred from Mutant Phenotype

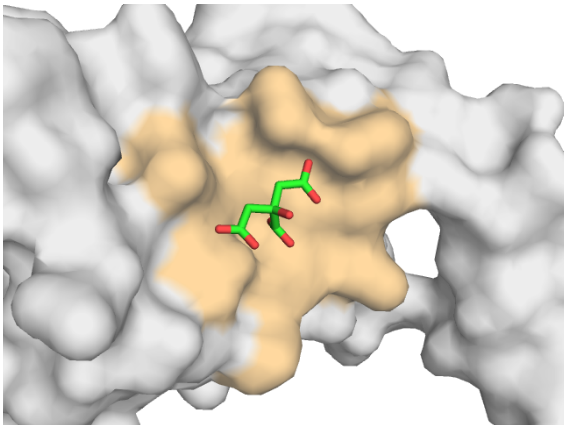

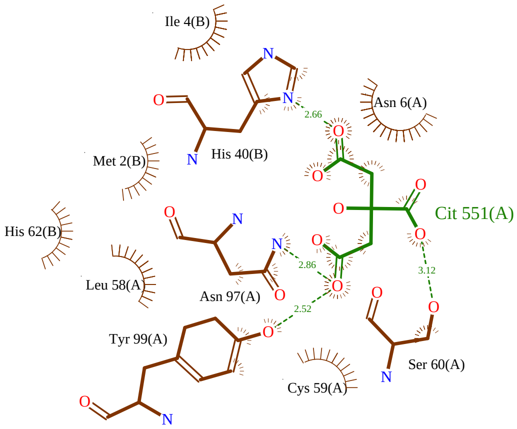

protein	amino acids contacts (binding site)									
PDB:1gcz:chainA:P14174	M	I	N	H	L	C	S	H	N	Y
PDB:1gd0:chainA:P14174	M	I	N	H	L	C	S	H	N	Y
sp:P14174:MIF_HUMAN	M	I	N	H	L	C	S	H	N	Y
tr:I4AY87:I4AY87_HUMAN	M	I	N	H	L	C	S	H	N	Y
sp:P30904:MIF_RAT	M	I	N	H	L	C	S	H	N	Y
tr:D3ZE63:D3ZE63_RAT	M	I	N	H	L	C	S	H	N	Y
tr:D4A3P7:D4A3P7_RAT	M	I	N	H	L	C	S	H	N	Y
sp:P34884:MIF_MOUSE	M	I	N	H	L	C	S	H	N	Y
tr:Q545F0:Q545F0_MOUSE	M	I	N	H	L	C	S	H	N	Y
tr:Q9U228:Q9U228_CAEEL	V	S	N	H	F	A	V	K	E	V
sp:Q18785:MIF2_CAEEL	M	R	A	E	V	I	S	K	T	H
tr:G3MU38:G3MU38_CAEEL	M	R	A	E	V	I	S	K	T	H

protein	whole protein ident	whole protein simil	domain-based ident	domain-based simil	contact-based ident	contact-based simil
PDB:1gcz:chainA:P14174	0.97	0.97	1.0	1.0	1.0	1.0
PDB:1gd0:chainA:P14174	0.97	0.97	1.0	1.0	1.0	1.0
sp:P14174:MIF_HUMAN	1.0	1.0	1.0	1.0	1.0	1.0
tr:I4AY87:I4AY87_HUMAN	1.0	1.0	1.0	1.0	1.0	1.0
sp:P30904:MIF_RAT	0.9	0.98	0.9	0.98	1.0	1.0
tr:D3ZE63:D3ZE63_RAT	0.86	0.96	0.86	0.96	1.0	1.0
tr:D4A3P7:D4A3P7_RAT	0.83	0.96	0.83	0.96	1.0	1.0
sp:P34884:MIF_MOUSE	0.9	0.97	0.89	0.97	1.0	1.0
tr:Q545F0:Q545F0_MOUSE	0.9	0.97	0.89	0.97	1.0	1.0
tr:Q9U228:Q9U228_CAEEL	0.33	0.78	0.33	0.78	0.2	0.48
sp:Q18785:MIF2_CAEEL	0.32	0.73	0.32	0.76	0.2	0.48
tr:G3MU38:G3MU38_CAEEL	0.27	0.62	0.32	0.76	0.2	0.48

2',3'-cyclic-nucleotide 3'-phosphodiesterase

Best gene implication in ageing for this target family came from gene P13233 annotated in UniProt release 2014_02. Annotation GO 7568 (aging) was Inferred from Expression Pattern

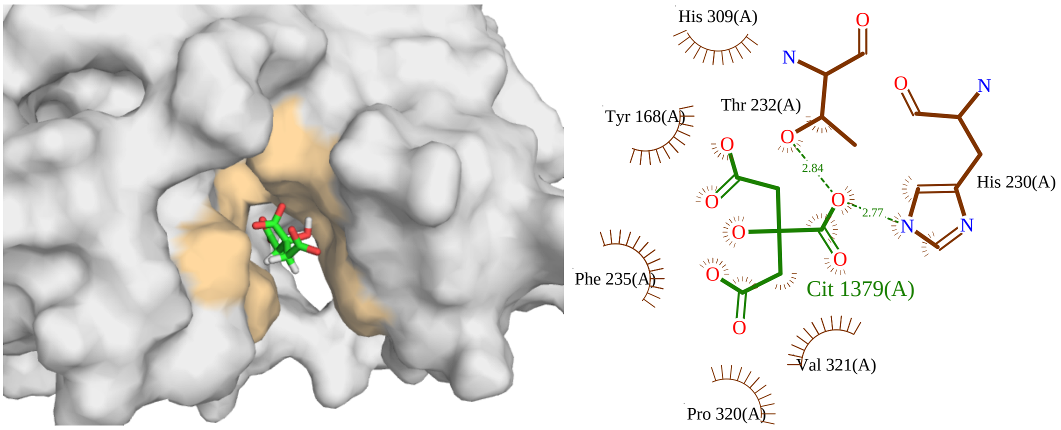

protein	amino acids contacts (binding site)						
PDB:2y1p:chainA:P16330	Y	H	T	F	H	P	V
sp:P09543:CN37_HUMAN	Y	H	T	F	H	A	V
sp:P13233:CN37_RAT	Y	H	T	F	H	P	V
tr:Q3TYL9:Q3TYL9_MOUSE	Y	H	T	F	H	P	V
sp:P16330:CN37_MOUSE	Y	H	T	F	H	P	V
tr:Q3TYV5:Q3TYV5_MOUSE	Y	H	T	F	H	P	V
tr:Q19804:Q19804_CAEEL	-	-	-	-	-	-	-

protein	whole protein ident	whole protein simil	domain-based ident	domain-based simil	contact-based ident	contact-based simil
PDB:2y1p:chainA:P16330	1.0	1.0	1.0	1.0	1.0	1.0
sp:P09543:CN37_HUMAN	0.85	0.96	0.8	0.95	0.86	0.96
sp:P13233:CN37_RAT	0.95	0.99	0.95	0.99	1.0	1.0
tr:Q3TYL9:Q3TYL9_MOUSE	0.95	0.95	1.0	1.0	1.0	1.0
sp:P16330:CN37_MOUSE	1.0	1.0	1.0	1.0	1.0	1.0
tr:Q3TYV5:Q3TYV5_MOUSE	1.0	1.0	1.0	1.0	1.0	1.0
tr:Q19804:Q19804_CAEEL	0.11	0.28	0.02	0.05	0.0	0.0

Caspase-7

Best gene implication in ageing for this target family came from gene F7F246 annotated in UniProt release 2014_02. Annotation GO 7568 (aging) was Inferred from Expression Pattern

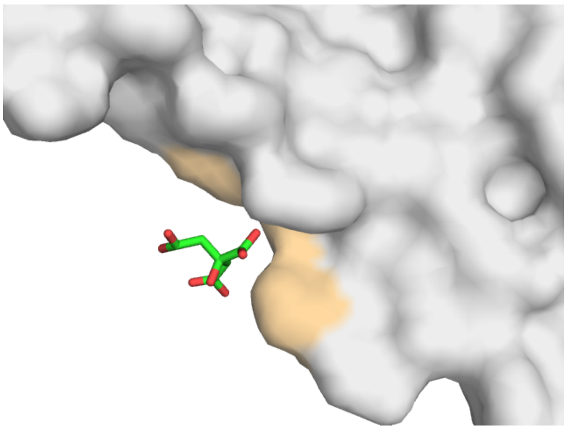

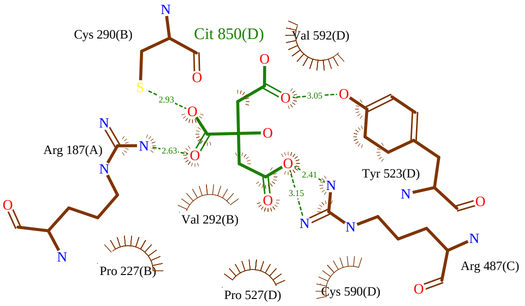

protein	amino acids contacts (binding site)			
PDB:2ql5:chainA:P55210	R	-	-	-
PDB:2ql5:chainB:P55210	-	P	C	V
PDB:2ql7:chainA:P55210	R	-	-	-
PDB:2ql7:chainB:P55210	-	P	C	V
PDB:2ql9:chainA:P55210	R	-	-	-
PDB:2ql9:chainB:P55210	-	P	C	V
PDB:2qlb:chainA:P55210	R	-	-	-
PDB:2qlb:chainB:P55210	-	P	C	V
PDB:2qlj:chainA:P55210	R	-	-	-
PDB:2qlj:chainB:P55210	-	P	C	V
sp:P55210:CASP7_HUMAN	R	P	C	V
tr:F7F246:F7F246_RAT	R	P	C	V
tr:Q4FJQ4:Q4FJQ4_MOUSE	R	P	C	V
sp:P97864:CASP7_MOUSE	R	P	C	V
sp:O02002:CASP1_DROME	Q	P	C	T
sp:O01382:ICE_DROME	Q	P	C	T

	whole protein		domain-based		contact-based	
protein	ident	simil	ident	simil	ident	simil
PDB:2ql5:chainA:P55210	1.0	1.0	1.0	1.0	0.25	0.0
PDB:2ql5:chainB:P55210	1.0	1.0	1.0	1.0	0.75	0.5
PDB:2ql7:chainA:P55210	1.0	1.0	1.0	1.0	0.25	0.0
PDB:2ql7:chainB:P55210	1.0	1.0	1.0	1.0	0.75	0.5
PDB:2ql9:chainA:P55210	1.0	1.0	1.0	1.0	0.25	0.0
PDB:2ql9:chainB:P55210	1.0	1.0	1.0	1.0	0.75	0.5
PDB:2qlb:chainA:P55210	1.0	1.0	1.0	1.0	0.25	0.0
PDB:2qlb:chainB:P55210	1.0	1.0	1.0	1.0	0.75	0.5
PDB:2qlj:chainA:P55210	1.0	1.0	1.0	1.0	0.25	0.0
PDB:2qlj:chainB:P55210	1.0	1.0	1.0	1.0	0.75	0.5
sp:P55210:CASP7_HUMAN	1.0	1.0	1.0	1.0	1.0	1.0
tr:F7F246:F7F246_RAT	0.84	0.95	0.89	0.97	1.0	1.0
tr:Q4FJQ4:Q4FJQ4_MOUSE	0.82	0.95	0.86	0.96	1.0	1.0
sp:P97864:CASP7_MOUSE	0.82	0.95	0.86	0.96	1.0	1.0
sp:O02002:CASP1_DROME	0.34	0.65	0.42	0.78	0.5	0.74
sp:O01382:ICE_DROME	0.34	0.67	0.43	0.79	0.5	0.74

Dcp-1 (FBgn0010501) associated phenotypes

cell death defective, decreased cell death, increased cell death, lethal - all die before end of pupal stage, neuroanatomy defective, neurophysiology defective, nutrition conditional, some die during pupal stage, starvation stress response defective, sterile

(Information from FlyBase)

Dcp-1 (UniProt:O02002) annotation

Function: Involved in the activation cascade of caspases responsible for apoptosis execution (By similarity). Proteolytically cleaves poly(ADP-ribose) polymerase (PARP). Loss of zygotic DCP-1

function causes larval lethality and melanotic tumors.
Subunit: Heterotetramer that consists of two anti-parallel arranged heterodimers, each one formed by a 22 kDa (p22) and a 13 kDa (p13) subunit.
Developmental stage: Present uniformly throughout embryos of stages 4 and 10. In stage 16 embryos, the expression becomes restricted to the central nervous system, the developing gonads, and a portion of the gut. In stage 17 embryos, expression is mainly localized in cells along the midline of the central nervous system.

(Information from UniProt)

Ice (FBgn0019972) associated phenotypes

cell death defective, decreased cell death, developmental rate defective, drug conditional, increased cell death, lethal - all die before end of pupal stage, male fertile, neuroanatomy defective, partially, partially lethal - majority die, radiation resistant, short lived, somatic clone, some die during pupal stage, tumorigenic

(Information from FlyBase)

Ice (UniProt:O01382) annotation

Function: Involved in the activation cascade of caspases responsible for apoptosis execution. Acts downstream of rpr. Cleaves baculovirus p35 and lamin DmO in vitro.
Subunit: Heterotetramer that consists of two anti-parallel arranged heterodimers, each one formed by a 21 kDa (p21) and a 12 kDa (p12) subunit. Inactive pro-form can homodimerize. Dronc and Drice can form a stable complex (PubMed:10675329). Interacts with Diap2 (via BIR3 domain) to form a stable complex (PubMed:18166655). (PubMed:10675329, PubMed:18166655).
Developmental stage: Expressed at all stages where apoptosis occurs.

(Information from UniProt)

Tyrosine-protein kinase ITK/TSK

Best gene implication in ageing for this target family came from gene P08630 annotated in UniProt release 2014_02. Annotation GO 8340 (determination of adult lifespan) was Inferred from Mutant Phenotype

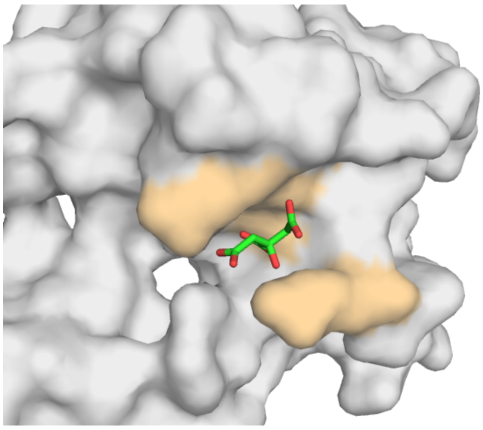

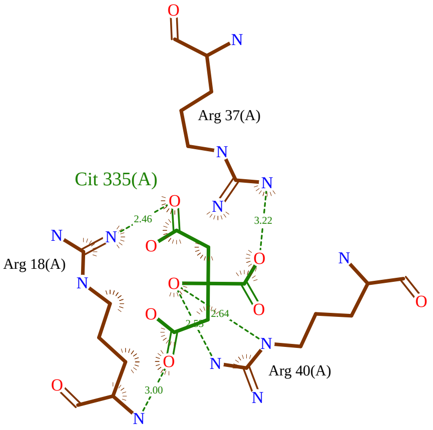

protein	amino acids contacts (binding site)
PDB:3s9k:chainA:Q03526	R R R T
sp:P42681:TXK_HUMAN	R R R T
sp:Q08881:ITK_HUMAN	R R R T
tr:Q501W1:Q501W1_RAT	R R R T
tr:D4A7W7:D4A7W7_RAT	R R R T
sp:P42682:TXK_MOUSE	R R R T
tr:B2RQ20:B2RQ20_MOUSE	R R R T
tr:A1A560:A1A560_MOUSE	R R R T
tr:Q5STT8:Q5STT8_MOUSE	R R R T
sp:Q03526:ITK_MOUSE	R R R T
sp:P08630:BTKL_DROME	R R S T

	whole protein		domain-based		contact-based	
protein	ident	simil	ident	simil	ident	simil
PDB:3s9k:chainA:Q03526	0.96	0.98	0.96	0.98	1.0	1.0
sp:P42681:TXK_HUMAN	0.43	0.66	0.52	0.84	1.0	1.0
sp:Q08881:ITK_HUMAN	0.93	0.97	0.91	0.96	1.0	1.0
tr:Q501W1:Q501W1_RAT	0.44	0.67	0.52	0.84	1.0	1.0
tr:D4A7W7:D4A7W7_RAT	0.98	0.99	0.99	0.99	1.0	1.0
sp:P42682:TXK_MOUSE	0.43	0.67	0.5	0.84	1.0	1.0
tr:B2RQ20:B2RQ20_MOUSE	0.43	0.67	0.5	0.84	1.0	1.0
tr:A1A560:A1A560_MOUSE	0.99	0.99	1.0	1.0	1.0	1.0
tr:Q5STT8:Q5STT8_MOUSE	0.99	0.99	1.0	1.0	1.0	1.0
sp:Q03526:ITK_MOUSE	1.0	1.0	1.0	1.0	1.0	1.0
sp:P08630:BTKL_DROME	0.34	0.61	0.42	0.72	0.75	0.74

Btk29A (FBgn0003502) associated phenotypes

cell shape defective, lethal - all die before end of first instar larval stage, mating defective, partially, short lived, size defective, some die during first instar larval stage

(Information from FlyBase)

Btk29A (UniProt:P08630) annotation

Function: Required for proper ring canal development. Also required for the development of male genitalia and for adult survival. (PubMed:10330180, PubMed:9655810).
Cofactor: Zn(2+)Note=Binds 1 zinc ion per subunit. ;
Subcellular location: Note=Ring canals.
Tissue specificity: Ring canals in the egg chambers and imaginal disks of third-instar larvae. (PubMed:10330180, PubMed:3110602, PubMed:9655810).
Developmental stage: Expressed both maternally and zygotically. Predominantly in early to middle embryogenesis, in larvae and adult females. (PubMed:10330180, PubMed:3110602).
Disruption phenotype: Flies exhibit shortened copulatory duration (due to incomplete fusion of the left and right halves of the apodeme that holds the penis during copulation) and reduced adult-stage life span. (PubMed:10330180).

(Information from UniProt)

Polyribonucleotide nucleotidyltransferase 1, mitochondrial

Best gene implication in ageing for this target family came from gene G3V6G7 via mapping the annotation from RGD 1307987 annotated in RGD 2014-03-11. Annotation GO subterm of 7568 (aging) was Inferred from Sequence Orthology

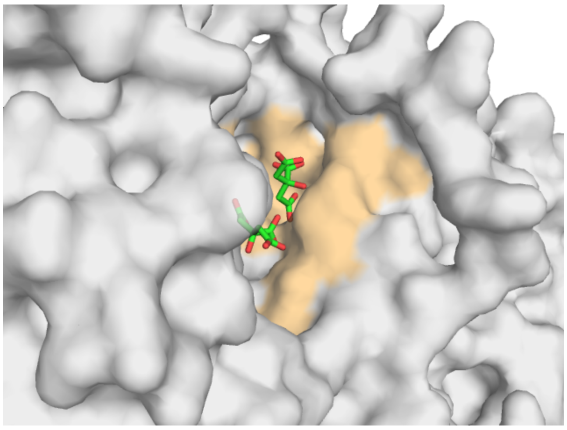

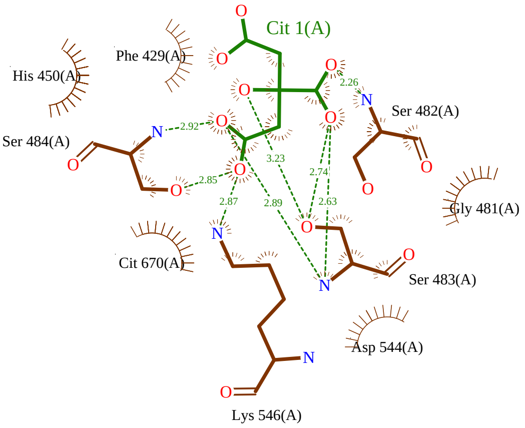

protein	amino acids contacts (binding site)
PDB:3u1k:chainA:Q8TCS8	R F R H G S S S D D K D
sp:Q8TCS8:PNPT1_HUMAN	R F R H G S S S D D K D
tr:G3V6G7:G3V6G7_RAT	R F R H G S S S D D K D
sp:Q8K1R3:PNPT1_MOUSE	R F R H G S S S D D K D
tr:Q8IH29:Q8IH29_DROME	R F R H G S S S D D K D
tr:Q95RX7:Q95RX7_DROME	R F R H G S S S D D K D
tr:A8JRH9:A8JRH9_DROME	R F R H G S S S D D K D
tr:Q9V9X7:Q9V9X7_DROME	R F R H G S S S D D K D
tr:Q5U1D1:Q5U1D1_DROME	R F R H G S S S D D K D
tr:Q965N3:Q965N3_CAEEL	R F R H G S S S D D K D

	whole protein		domain-based		contact-based	
protein	ident	simil	ident	simil	ident	simil
PDB:3u1k:chainA:Q8TCS8	1.0	1.0	1.0	1.0	1.0	1.0
sp:Q8TCS8:PNPT1_HUMAN	1.0	1.0	1.0	1.0	1.0	1.0
tr:G3V6G7:G3V6G7_RAT	0.92	0.97	0.94	0.98	1.0	1.0
sp:Q8K1R3:PNPT1_MOUSE	0.91	0.97	0.94	0.99	1.0	1.0
tr:Q8IH29:Q8IH29_DROME	0.48	0.76	0.54	0.79	1.0	1.0
tr:Q95RX7:Q95RX7_DROME	0.53	0.79	0.62	0.89	1.0	1.0
tr:A8JRH9:A8JRH9_DROME	0.48	0.76	0.54	0.79	1.0	1.0
tr:Q9V9X7:Q9V9X7_DROME	0.53	0.82	0.62	0.89	1.0	1.0
tr:Q5U1D1:Q5U1D1_DROME	0.53	0.82	0.62	0.89	1.0	1.0
tr:Q965N3:Q965N3_CAEEL	0.38	0.75	0.47	0.81	1.0	1.0

WBGene00015266 associated phenotypes

lethal, sterile

(Information from WormBase)
